# TinkerCell: modular CAD tool for synthetic biology

**DOI:** 10.1186/1754-1611-3-19

**Published:** 2009-10-29

**Authors:** Deepak Chandran, Frank T Bergmann, Herbert M Sauro

**Affiliations:** 1 Department of Bioengineering, University of Washington, Box 355061, William H. Foege Building, Room N210E, Seattle, WA, 98195-5061, USA; 2 Keck Graduate Institute, 535 Watson Drive, Claremont, CA, 91711, USA

## Abstract

**Background:**

Synthetic biology brings together concepts and techniques from engineering and biology. In this field, computer-aided design (CAD) is necessary in order to bridge the gap between computational modeling and biological data. Using a CAD application, it would be possible to construct models using available biological "parts" and directly generate the DNA sequence that represents the model, thus increasing the efficiency of design and construction of synthetic networks.

**Results:**

An application named TinkerCell has been developed in order to serve as a CAD tool for synthetic biology. TinkerCell is a visual modeling tool that supports a hierarchy of biological parts. Each part in this hierarchy consists of a set of attributes that define the part, such as sequence or rate constants. Models that are constructed using these parts can be analyzed using various third-party C and Python programs that are hosted by TinkerCell via an extensive C and Python application programming interface (API). TinkerCell supports the notion of a module, which are networks with interfaces. Such modules can be connected to each other, forming larger modular networks. TinkerCell is a free and open-source project under the Berkeley Software Distribution license. Downloads, documentation, and tutorials are available at .

**Conclusion:**

An ideal CAD application for engineering biological systems would provide features such as: building and simulating networks, analyzing robustness of networks, and searching databases for components that meet the design criteria. At the current state of synthetic biology, there are no established methods for measuring robustness or identifying components that fit a design. The same is true for databases of biological parts. TinkerCell's flexible modeling framework allows it to cope with changes in the field. Such changes may involve the way parts are characterized or the way synthetic networks are modeled and analyzed computationally. TinkerCell can readily accept third-party algorithms, allowing it to serve as a platform for testing different methods relevant to synthetic biology.

## Background

Systems level modeling of biological systems has provided mathematical explanations for experimental observations [1-3]. One of the consequences of such understanding has been the ability to design and engineer synthetic biological networks inside cells, which has given rise to the field of Synthetic Biology [4]. The idea of engineering biological systems brings together concepts from various fields. While the laboratory procedure at present is borrowed from genetic engineering, concepts such as abstraction and interchangeable parts are taken from computer science and electrical engineering. Synthetic biology introduces the notion of biological "parts" [4], which are individual components that can be assembled in different ways to construct synthetic networks with different functions. Networks built by different engineers can then be reused to construct larger networks [5], much like a programmer using existing subroutines to build a new program more efficiently. Since synthetic biology is a young field, the best practices for making molecular biology interchangeable and programmable have not been established. Nonetheless, various success stories [6-11] have shed light on the enormous potential of synthetic biology to understand fundamental science [4] or create new solutions for applications ranging from fuels [12] to medicine [13].

The terminology, software, and laboratory procedures required to push synthetic biology forward are still in development. However, one can anticipate that certain key concepts such as standardization and modularity will become commonplace in synthetic biology [14]. In synthetic biology, the need for standardization exists at different levels. At one level, there is a standard for defining parts. While biological parts are the individual components for building a network, *standardized *parts contain additional restrictions that are intended to make synthetic networks easier to build, more reliable, and easier to exchange. An existing standard is the standard assembly [15], which has made DNA assembly simpler. In future, it is anticipated that standards will also exist for describing the dynamics of a part; for example, standard promoter parts might contain a "strength" value, describing its efficiency in recruiting RNA polymerase under some standard environmental condition [16]. This leads to the second level of standards, which describes parts in a computer-readable format such as the Resource Definition Language [17,18], so that searching and organizing parts can be automated. Under such a framework, parts could be organized by a defined ontology. The third level of standards applies to computational models. While there are existing standard formats for representing biological models [19,20], synthetic biology models might require additional information such as the DNA sequence or specific information about the parts that are needed to physically construct the network.

Several issues in modeling for synthetic biology have to be addressed. First, a complete model should support information for computational analysis as well building the physical biological circuit. Second, due to the fact that synthetic biology spans multiple disciplines, a large number of analyses may be possible on synthetic networks, ranging from dynamical systems analysis to analysis of the DNA sequence or statistics on the part usage in the model. The software application presented in this work, TinkerCell, will address these issues. While TinkerCell targets synthetic biology, it addresses several issues that are equally applicable outside the field. These issues include support for third-party libraries, modular design of networks, flexible modeling framework, and flexible visual format. TinkerCell is an improvement on a similar effort, Athena, by the same authors [21]. Athena addressed issues such as modular design of networks and biological parts. However, the underlying design of Athena was not as flexible as it was intended to be. For example, Athena was not able to support an ontology of biological parts. Due to such limitations in the design, the project was completely restarted under a different name.

### Existing computational tools

Numerous software tools exist that allow construction and analyses of models using scripts or visual interfaces. A comprehensive list can be found at . Some of these applications include Jarnac [22], JDesigner [22], CellDesigner [23], Bio-Tapestry [24], PySCeS [25], BioJADE [26], little-B [27], SynBioSS [28], ProMot [29-31], and Antimony [32]. Each of the applications have their respective advantages. For example, Jarnac and PySCeS are highly flexible due to their programming interface. Little-B is similar, building on Lisp, but it supports modules in addition. CellDesigner offers a plug-in interface, which has permitted the community to add new features. BioTapestry has a simple visual depiction for genetic networks and genetic modules. BioJADE and SynBioSS, being synthetic biology applications, support a parts database [33]. Antimony [32] is a C/C++ library and an editor for defining modular models using a human-readable script. ProMot is similar but also has a visual interface that supports modularity as well as using parts to compose a circuit [30]. We considered that TinkerCell should, at the least, contain the qualities of each, which include the flexibility of programming, plug-in support, database support, modularity, and ability to construct genetic networks.

However, the intent behind TinkerCell is not to create yet another modeling application, but to create an application that can serve as a host to the algorithms, data, and ontologies that the synthetic biology and the systems biology community can offer. Unlike most modeling applications, TinkerCell does not impose a particular modeling method (e.g. differential equations), visual representation, or a strict definition of a model. It has a very generic representation of a network, and the algorithms that use the information provide the interpretation.

## Results and Discussion

**Figure 1:**
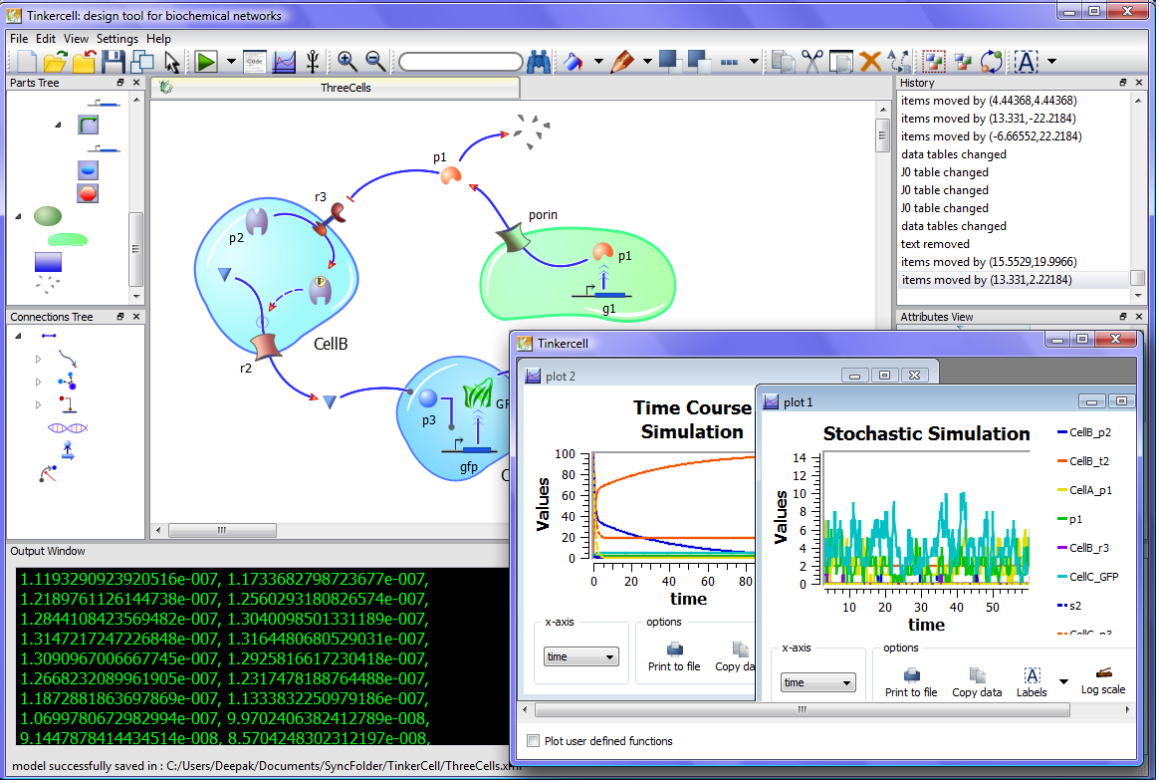
**Screenshot of TinkerCell**. This is a screenshot from TinkerCell showing a system involving three cells. Models are constructed using components available in the parts catalog, which is located at the top left. Some of the components used in this system are cells, membrane proteins, fluorescent proteins, small molecules, and genes. Below the parts catalog is the catalog of reactions, which includes reactions such as enzyme catalysis and transcriptional regulation. A history window is located at the top right. The plot window can hold multiple plots, including 3D surface plots.

The application named TinkerCell is a synthetic biology CAD tool for visually constructing and analyzing biological networks (see Figure 1). The visual interface alone does not have any analysis capabilities. The analyses are performed by third-party C or C++ libraries and Python modules that interface with TinkerCell. Therefore, TinkerCell can be thought of as a front-end to C and Python programs.

The target audience for TinkerCell fall into three categories: the users, the collaborators, and the developers. The users would construct models in TinkerCell using the visual or scripting interface, access parts from database(s), and analyze the model using numerous available functions. The collaborators provide functions focusing on specific tasks. For example, one collaborator who hosts an *E. coli *database might add a Python script to TinkerCell for accessing that database. Another collaborator might add a complex algorithm for analyzing the stability of models. The collaborators benefit from TinkerCell because they can use it as a medium for presenting their tools to the community. The developers are experienced programmers who wish to add new interfaces or novel features to TinkerCell. Alternatively, the developers may also use the TinkerCell Core library to construct a new software application. The users benefit from both the collaborators and developers by being able to use the functions and features that they provide.

### Features

Depending on the interests of the individual, different features of TinkerCell may be more important than others. The next five sections describe four primary categories of features that are provided by TinkerCell.

#### Modeling and analysis

TinkerCell provides visual and script-based modeling interface, which uses Antimony scripts [32]. The visual interface is slightly more generic than the script interface and provides default equations for transcriptional rates and other reactions. The models can also contain compartments, which are physically separated regions of a system.

Numerous functions are available in TinkerCell for analyzing a constructed model. These include deterministic [34] and stochastic simulation algorithms [35], metabolic control analysis [25], structural analysis [25], flux-balance analysis [36], one-dimensional and two-dimensional steady state analysis, and centrality measurements [37].

#### Third-party functions and scripts

TinkerCell provides a convenient way for users to integrate their functions with TinkerCell's user interface. The functions listed at the end of the previous section are not built-in functions. They are provided as plug-ins, or features that can be added to TinkerCell without modifying the existing program. Users with knowledge of the C or Python languages can add more functions to TinkerCell. This is done by either placing compiled C libraries in the Plug-ins/C folder or listing Python scripts in the "python-scripts.txt" text file; TinkerCell will automatically display these functions in its menu of functions.

The ability to readily add new functions will allow TinkerCell to be used as a means of contributing new functions to the community. These functions may range from mathematical analysis to database support or sequence analysis.

#### Synthetic biology

Models in TinkerCell are constructed using "parts" from a catalog. This catalog is defined externally in an Extensible Markup Language (XML) file; in future, this XML file will be replaced by a catalog from a parts database. Each part in the model can store a large amount of information associated with the part, such as database IDs, annotation, ontology, parameters, equations, sequence, and information required by experimentalists, such as plasmid information or restriction sites found within the part. Parts can be loaded along with all their known information from databases, although this feature depends on the growth of the databases themselves.

Being able to store information about each part permits operations such as loading of parts from a database and testing how a particular part affects the model. In contrast, a conventional modeling application that solely uses variables, equations, and parameters to describe a model will have difficulty replacing a single part, because it would need to identify the parameters and equations associated with that part. Applications that do utilize a database often limit the number of parameters in the model [28], thus sacrificing some flexibility. TinkerCell achieves the same without compromising flexibility.

Since the current synthetic networks are mostly composed of genetic networks, TinkerCell has extensive support for constructing gene regulatory networks by connecting parts such as promoters, ribosomal binding sites (RBS), and other genetic components listed in the parts catalog. When parts are connected together, TinkerCell will automatically derive rate equations [38] for transcription and translation by looking at the transcription factors bound to operator sites. The RBS part's parameters are also automatically utilized in the translation rate.

Python and C functions are available for getting and setting information about the parts. These functions can be used by third-party scripts to export information needed by experimentalists [17,18] as well as validating the model: for example, one script can check whether the plasmid containing each part have compatible antibiotic resistance genes and restrictions sites and suggest changes. Due to limited data, such features are not present in TinkerCell, but TinkerCell's design is ideal for incorporating such features.

#### Modularity

TinkerCell supports the ability to construct new models by connecting existing models. This is achieved by encapsulating a model as a "module". Modules contain interfaces that allow them to "connect" to other modules. This idea is borrowed from electric engineering, where complex circuits are created by connecting modules that provide specific functions. How the concept of modularity maps to biological systems is an open question [39,40], but by supporting this idea, TinkerCell can serve as a platform for experimenting with the concept of modularity.

#### Plug-in interface and Core library

TinkerCell is extensible at different levels. At one level, users can add new Python and C functions to the menu of functions available in TinkerCell. At a lower level, programmers can make more significant additions by writing new plug-ins. For example, the menu of C and Python functions itself is a plug-in. Similarly, the rate equations and modeling framework is controlled by a plug-in. By adding new plug-ins, it is possible to introduce different modeling methods and new visual interfaces in TinkerCell. Users who wish to use optional plug-ins may download them into their TinkerCell Plugins folder. Detailed documentation on the plug-in framework is available at , under the "Design and framework" link.

## Methods

TinkerCell is organized in layers, with the bottommost layer being a generic API for constructing network drawing programs and the top-most layer being a scripting interface. TinkerCell is extensible at each of the layers. There is an additional level of flexibility provided by the XML files that load the ontology of parts and connections. The goal of this structure is to foster contributions from the community.

### The Core library

**Figure 2:**
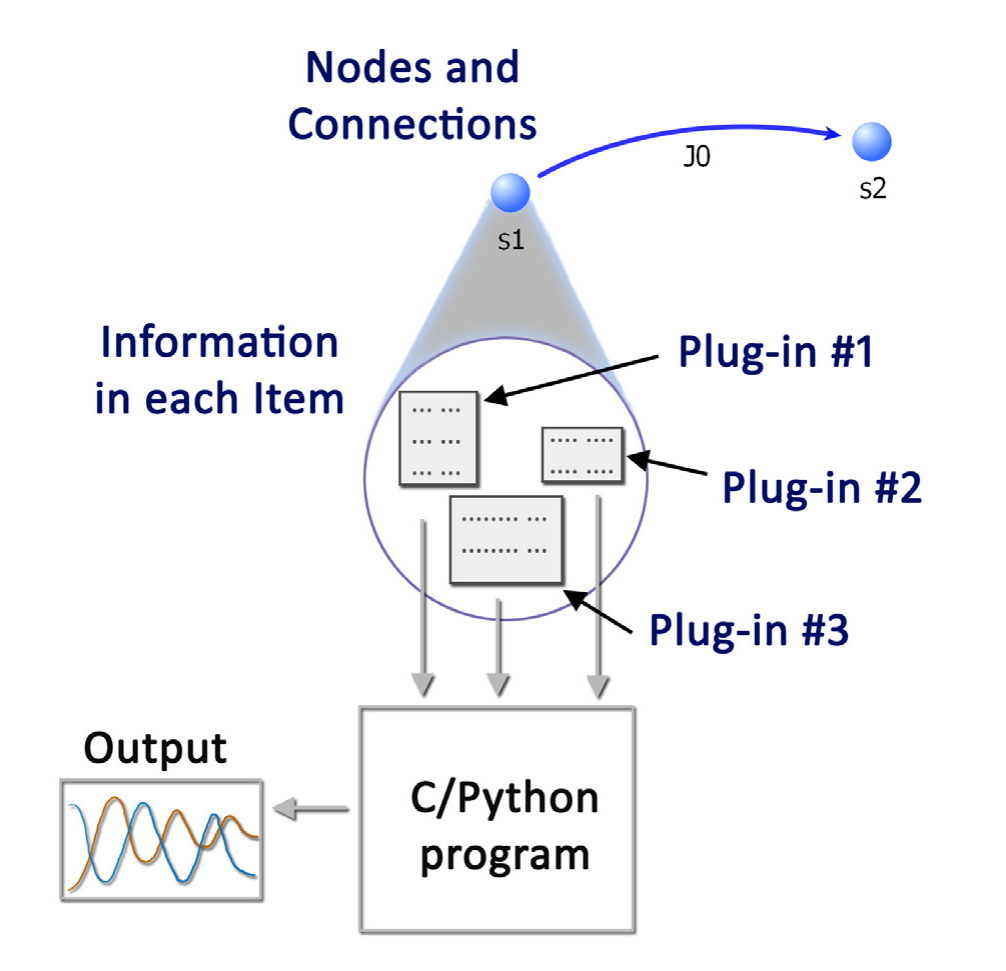
**The TinkerCell Core API**. Shown here is the basic structure provided by the Core library. The Core library defines data structures and functions for drawing nodes and connections. Each node and connection can contain "data" associated with that node or connection. This data can include information such as parameters, equations, DNA sequence, or database IDs. Plug-ins, both C and C++, and Python scripts are responsible for populating the data inside each node and connection. For example, a C++ plug-in is responsible for adding the a table of parameter values to each node and connection; another plug-in is responsible for adding reaction rates to all the connections. Another small plug-in stores and updates the spacial co-ordinates of all cells in the model, allowing a modeler to use cell position information inside the model. C and Python programs utilize all this information to perform various analyses, including simulations. Plug-ins also provide the graphical interface for viewing and editing the information stored inside the items.

The TinkerCell Core library is a C++ library built using the Qt Toolkit 4.5.0 [41]. The Core library contains the data structures and functions for drawing parts and connections and storing information associated with those items (see Figure 2). It provides an API which can be used to build new network drawing programs. The Core library also provides support for C functions and a generic console window, which is currently used as a Python console but can later be used to support other languages as well. The complete TinkerCell application is a collection of plug-ins that utilize the TinkerCell Core library in order to provide the full set of features. Throughout this article, the name "TinkerCell" will refer to the collection of plug-ins and the TinkerCell Core library, because the Core library alone has no modeling capability. Extensive documentation on the Core library is available at . A small example of using the Core library to construct a new program is also provided with the TinkerCell source code.

### Plug-ins and flexible modeling framework

TinkerCell uses the notion of plug-ins. Plug-ins are C++ programs that add new features to TinkerCell without altering the existing code, thus allowing programmers to extend TinkerCell. The current set of plug-ins are responsible for storing parameters, rate equations, and other information relevant to modeling as well as storing sequence and other details needed for physical construction of the network. The plug-ins also provide the user interface for editing these values. The complete TinkerCell application is composed of a collection of plug-ins that build on the TinkerCell Core library. Plug-ins are responsible for providing visual features such as scaling and coloring as well as modeling features such as loading the catalog of parts and connections and inserting parts and connection from that catalog.

An important role of plug-ins is defining what information can be stored in TinkerCell models. For example, the Numerical Attributes plug-in and the Stoichiometry plug-in, together, ascertain that all TinkerCell models will contain sufficient information to generate the stoichiometry matrix and rate equations, which are required for generating differential equations and stochastic simulations. Additional information, such as function definitions and events, are added by other plug-ins.

While the existing plug-ins focus on building models based on stoichiometry and rates, it is possible to write new plug-ins that focus on other modeling approaches, such as rule-based or Boolean modeling. The current set of plug-ins do not include such approaches, but the underlying structure of Tinker-Cell is not limited to any one modeling approach.

### C and Python interface

**Figure 3:**
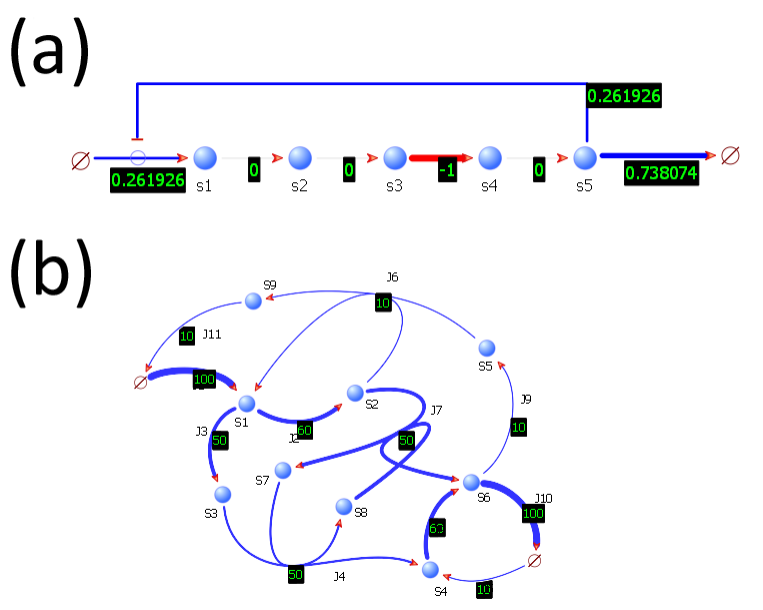
**Graphical output from C/Python programs**. Shown here are the outputs from two functions written in Python and C, respectively. The screenshot in (a) shows the output from a Python script that converts TinkerCell models into PySCeS [25] models and uses PySCeS to compute the control coefficients. The script adjusts the line widths and colors in the visual model accordingly. Negative control coefficients are colored red and positive ones are colored blue. The screenshot in (b) is the output from the flux balance analysis function in TinkerCell. The function is a C program that gets the stoichiometry matrix and constraints from TinkerCell and uses LPSolve C library [36] for linear programming. The program then sets the line widths of the reactions in the visual model according to the output from LPSolve.

**Figure 4:**
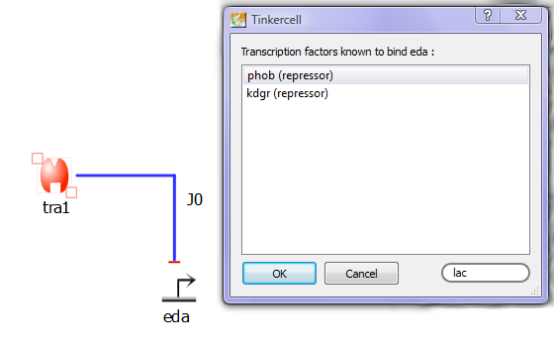
**Accessing RegulonDB**. This screenshot shows the interface provided by the Python script that searches RegulonDB [44] and provides the user with a list of parts from *E. coli*. The script uses the selected part's family information to identify it as a promoter, RBS, coding sequence, or transcription factor. It then uses this information to search the appropriate types of parts in RegulonDB. Additionally, the script looks at the connections made by the part to prune the list. For example, if a transcription factor is regulating the selected promoter, then the program will only list the promoter sites that are regulated by the particular transcription factor. For later reference, the script also stores the RegulonDB ID and other information within the parts.

Plug-ins that are related to modeling are almost always supported by C or Python functions. For example, the Numerical Attributes plug-in and the Stoichiometry plug-in enrich the model with sufficient information to generate dynamic models, but the plug-ins themselves do not perform the analysis. Instead, each plug-in provides an API containing functions such as "getParameter", "getRate", or "getStoichiometry", allowing third-party C and Python programs to obtain the necessary information needed to carry out a simulation or other analysis. Almost every plug-in in TinkerCell exposes an API, creating a rich C and Python API with over a hundred different functions. The collection of API functions allow C and Python programs to get or set kinetic information such as parameters, rates, functions, and stoichiometries, or change visual aspects such as positions, colors, and line widths (see Figure 3). The API also provides ways for C and Python programs to bring up dialogs, asking for user inputs (see Figure 4).

All C and Python programs automatically run on separate threads, allowing a user to continue working on a model while a time consuming task is running. TinkerCell's default simulators compile the model as a separate C program, allowing the simulations to run at the speed of a compiled C program. This strategy is used by the deterministic simulator as well as the stochastic simulator, where the speed gain is more visible.

The C API has been used to include the following C programs in TinkerCell: deterministic simulation and steady state analysis using the Sundials library [34], stochastic simulation using a custom C library, flux balance analysis using LPsolve [36], and custom C programs that automatically modify reaction kinetics and calculate loops in the Jacobian matrix. The Python API has been used to include the SciPy module [42], PySCeS module [25], NerworkX module [37], custom programs for generating transcriptional rate formulas, and another custom program for generating FASTA files for the DNA parts in the model.

A few example Python scripts for interfacing with TinkerCell are provided (see Additional File 1).

### Modular design of networks

**Figure 5:**
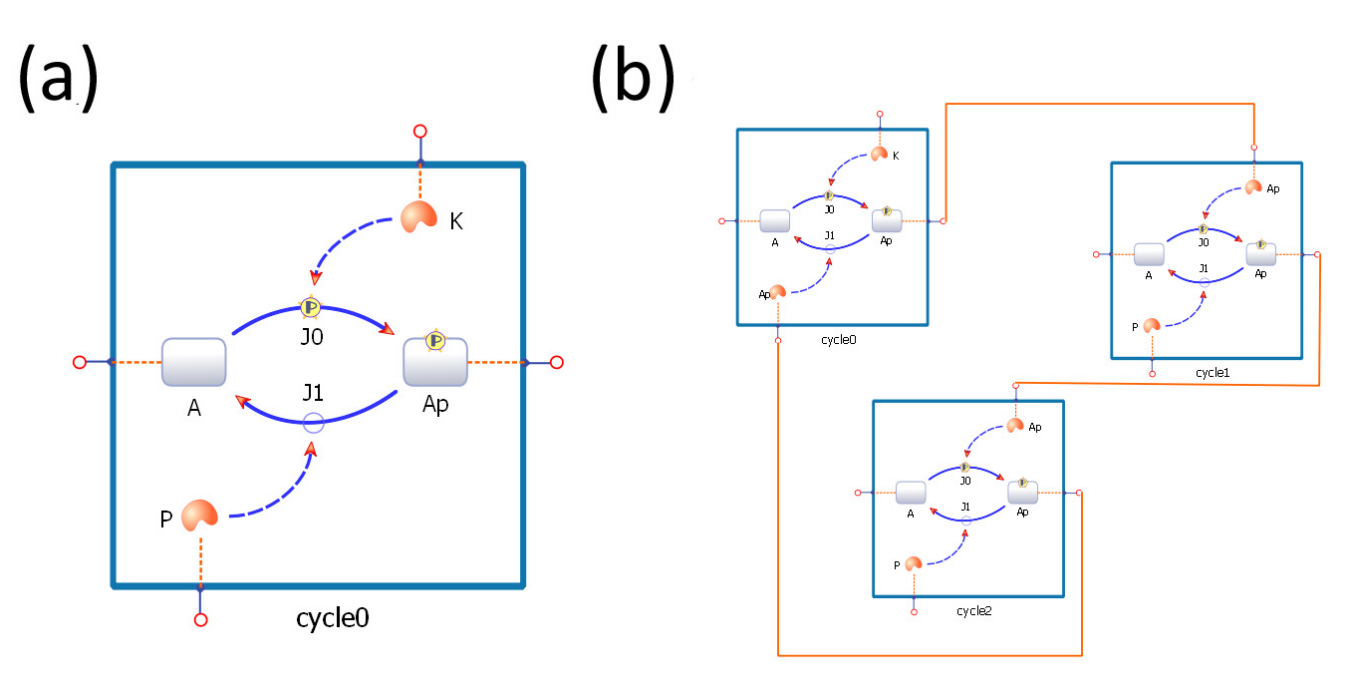
**Connecting modules**. Figure (a) shows a simple phosphorylation and dephosphorylation cycle. The cycle is converted to a module by placing all the components inside a module box and declaring the proteins as interfaces. The interface items are shown as small pins around the module box. The interface items can be used to connect one module to another, as shown in (b). In figure (b), the module shown in (a) is copied and pasted three times. The three modules are connected together to form an oscillating protein network [43]. The orange connections between the modules' interface pins indicate that the connected components are the same. For example, the phosphorylated protein in the first module is the molecule as the kinase in the second module. In other words, modules are connected by defining the shared components.

In TinkerCell, new models can be constructed by connecting existing networks to one another. Such composable networks, or modules, are constructed by defining interfaces. A user may declare one or more components of a module as interfaces. These interfaces can then be connected with one another, which indicates that the respective components of the modules have been merged. For example, if a user wishes to construct a cascade of phosphorylation cycles [43], then they may simply connect the kinase of one module to the phosphorylated protein in the other module. This connection indicates that the kinase in the first module and the phosphorylated protein in the second module are both the same molecule. Two species that are merged become the same physical entity (see Figure 5). The connections do not alter the modules themselves, thus the modules are reusable.

#### Genetic modules

**Figure 6:**
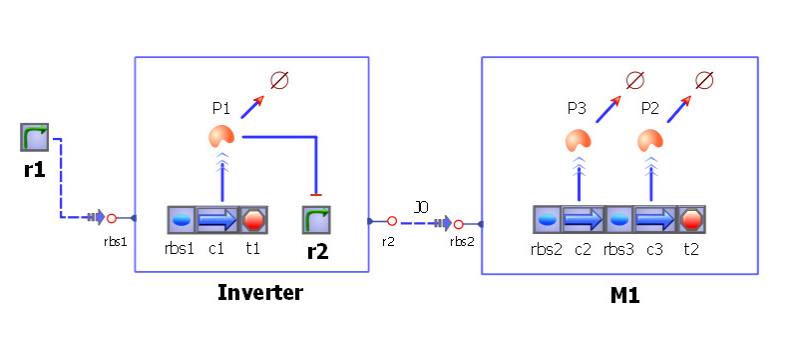
**A genetic module**. Shown here is a genetic module as they are often used in the synthetic biology community. The first module, the Inverter, is usually placed under the control of some promoter, r1. When r1's activity is high, r2 is low, and when r1 is low, r2 is high, hence the inversion. The dotted line between promoter r1 and the Inverter module can be thought of as the flux of RNA polymerase. The connection between the Inverter module and the M1 module is also the same. Therefore, r2 controls the transcription rate of the genes in module M1.

Another way to connect two or more modules is by introducing new reactions between the interfaces. The synthetic biology community is familiar with the notion of modules in the context of DNA. For example, if one module is responsible for regulating a promoter and the next module is situated downstream of the promoter, then the first module regulates the second through its promoter. In Tinker-Cell, this can be accomplished by connecting the promoter of the upstream module to the gene in the downstream module (see Figure 6). This connection belongs to a special family of connections that represents RNA polymerase activity along a DNA strand, known as "PoPS" (Polymerase Per Second [16]) in the synthetic biology community. The synthetic biology community often uses other acronyms such as "RiPS" (Ribosomes Per Second), which can also be modeled similarly.

#### Modules as composite parts

The synthetic biology community is familiar with the notion of a "composite part", or a biological part that has been constructed by assembling other parts together [33]. TinkerCell can represent such an object as a module. Each sub-part inside the composite part retains its original set of attributes and other information while the composite part can have its own family and set of attributes. The C and Python API can be used to access the parameters, annotation, authorship, and list of sub-parts.

Just as with any other component in a model, modules can have rate expressions describing their input and output behavior. This allows a modeler to describe the behavior of a composite part is an abstract way without being forced to know all the internal details.

### Support for standards

It is our anticipation that the growth in synthetic biology will result in a general agreement in the community about what attributes are needed to define a biological part. For example, a promoter part might be defined by its DNA sequence, operator sites, and binding affinity of RNA polymerase. The last parameter has a physical interpretation, therefore models using this parameter of the promoter part will be more coherent: the parameters used in the model will have a standard meaning that is understood by the community rather than solely by the model designer. In contrast, when a model uses parameters defined by the model designer, the physical interpretations of those parameters may not be clear. When all the parameters and rate equations in a model have clear interpretations, it becomes possible to carry out automated procedures, such as finding real parts that fit the model. The next three sections will describe how this idea of standard parameters is supported, but not enforced, in TinkerCell.

#### Parts ontology

**Figure 7:**
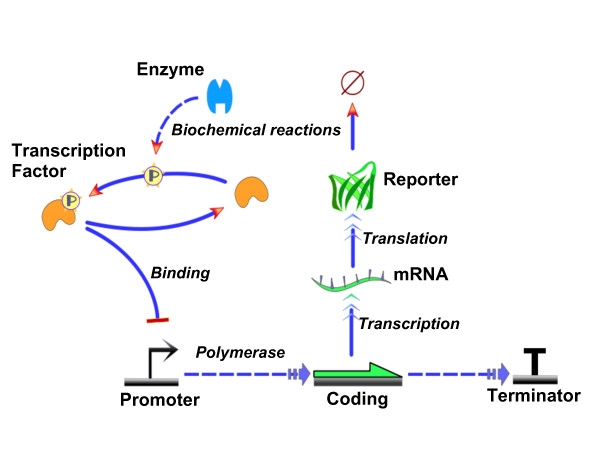
**Each part and connection belongs to a family**. Each item in TinkerCell belongs to a family. Shown here is a screenshot from Tinkercell with labels indicating the family that each part or connection belongs with. A family structure is important if a model should support ontologies or interact with databases. For example, the family information can be used by a Python script to screen for all the RBS parts being used in the model. The default visual representation can be changed by clicking the mouse right-button on the family icon. Each connection family has a different arrowhead, which can also be replaced.

Ontologies or other ways of organizing biological parts will become necessary when annotating a model. Every item in TinkerCell belongs to a "family" (see Figure 7). The families themselves are defined in an external XML file and are organized as a graph structure. For example, the family named "Transcription Factors" is a subset of the family named "Proteins". Each family is defined with a set of attributes. For example, genes contain a "sequence" attribute. In addition to sequence, promoters or RBS contain a "strength" attribute, which is a number describing the relative strength of the promoter or RBS. The hierarchy of parts will be obtained from a database of biological parts in future.

#### Reactions ontology

Similar to the part families, every connection between parts in TinkerCell is also identified with a family. Examples of connection families are "Biochemical reaction", "Binding reaction", and "Transcriptional regulation". Each connection family also contains attributes; for example, "Transcription regulation" contains parameters named "h", the Hill co-efficient, and "Kd", the dissociation constant. These attributes define the standard parameters required to characterize the dynamics of the reaction. Models that use such standard parameters would be easier to interpret and carry out automatic operations because the parameters have a well understood interpretations that are independent of the modeler. The connection families are also loaded from an external XML file and will be obtained directly from a database in future.

#### Using ontologies in models

**Figure 8:**
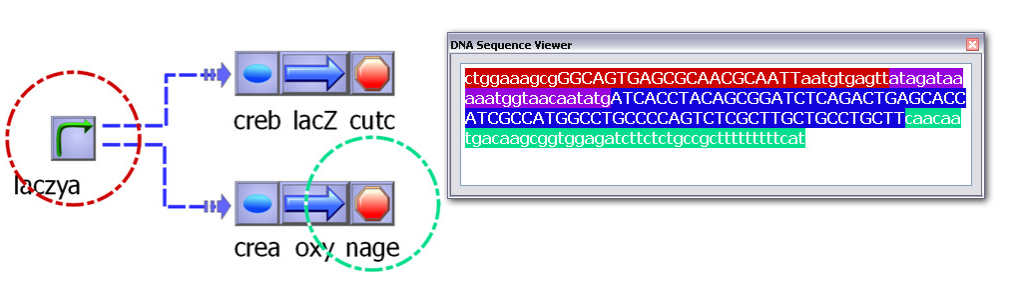
**Viewing DNA sequence**. In the screenshot above, several biological parts are connected together. However, the promoter named "laczya" is regulating two other gene segments. The regulation is shown by the two dotted arrows. Since the promoter is regulating two genes, it is implied that there are two copies of this promoter, one upstream of each gene that it regulates. This is one method of showing regulation in an abstract way in the synthetic biology community [16]. The color coded circles reflect the first and last parts in the displayed sequence. The individual sequences are loaded from RegulonDB [44] via a Python script. The sequence attribute is only available for items of specific families, as described by the family tree of biological parts. The sequence viewing window is provided by a small plug-in.

The advantage of constructing models that also define the family of each item is that plug-ins or third-party programs can utilize the fact that certain parts or connections will always have certain parameters. An example is the "Hill equations" Python script, which automatically generates rate equation using the fractional saturation model [38]. The script utilizes the fact that every "Transcription regulation" connection contains a "Kd" and a "Hill" parameter, and it uses these parameters to automatically generate the transcription rate equation. Another Python script included with TinkerCell searches the RegulonDB database [44] for RBS, promoters, and terminators from *E. coli *and automatically fill in appropriate attributes based on the type of part that is selected; for example, when the script sees a promoter, it adds the sigma factor information to the part, and when it sees a terminator, it adds information about whether or not it is a rho-dependent terminator. An example plug-in that uses the family information is the DNA sequence viewer (see Figure 8), which assumes that every item of the family "DNA" will contain an attribute called "sequence" and is able to present the information visually. One can imagine other functions where features of the promoter, such as the sigma-factor, may be used within the model to provide additional details, and the user can load different promoters from a catalog of biological parts to test how the different promoters affect the network.

It should be noted that the families and their attributes are not defined within TinkerCell, so the algorithms are general for any future ontology that the synthetic biology community will adopt. In addition to the family information, each part and connection in TinkerCell contains its own annotation, storing information such as authorship, references, and date. As with any other information in Tinker-Cell, all of this information is accessible and editable from C and Python.

#### Visual formats

Standard visual formats, such as the standard symbols that are used to draw electronic circuits, allow network diagrams to be unambiguous. While systems biology has made progress to standardize visual representations of biological systems [45], such standards for synthetic biology are still in development [46]. TinkerCell has been designed so that it will be able to cope with a variety of visual standards or even multiple visual standards. This is achieved by having a flexible visual representation. The visual representation of proteins, genes, promoters, and the rest of the parts are stored as XML files. These files are generated using a polygon drawing program that comes with TinkerCell. The same file format is used for arrowheads and decorators such as phosphorylation sites, which keeps visual representations for those objects flexible as well.

In order to cope with a future visual standards, new files will be generated using the polygon drawing program that match the prescribed visual standard.

### Genetic networks

Since the majority of the current synthetic networks are genetic networks, TinkerCell gives special consideration to them. TinkerCell provides three different ways for modeling genetic networks, allowing the modeler to choose which is most suitable. This builtin support for multiple modeling frameworks reflects TinkerCell's underlying modeling framework, which is independent of the modeling technique.

### Fractional saturation models

**Figure 9:**
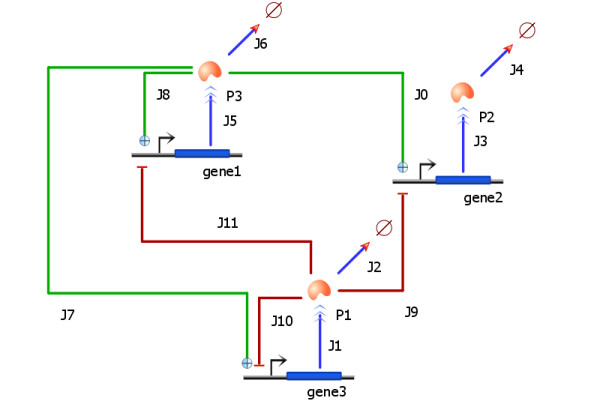
**Simple method of modeling gene regulatory networks**. Shown here is an oscillatory gene regulation network [9]. The rate equations in this model assume that the transcription factor association and dissociation from the operator sites are at equilibrium. Under this assumption, the fractional saturation model [38] can be used to determine the rate of production of each gene product. The arrowheads are indicative of the type of reaction. For example, arrow from the genes to the proteins have a special arrowhead indicating that it is a transcription reaction. The mRNA step can be included automatically if needed from the context menu (mouse right-click).

The first method of modeling gene regulatory networks uses equilibrium assumption for transcription factor binding [38]. Under this model, a single rate expression is generated that captures the probability of the promoter being in an active state. This rate expression assumes that the transcription factor binding and unbinding are at equilibrium. TinkerCell automatically generates the rate expressions when a user connects transcription factors to a gene. Figure 9 shows an oscillatory network constructed using this framework.

#### Using parts to model genetic networks

**Figure 10:**
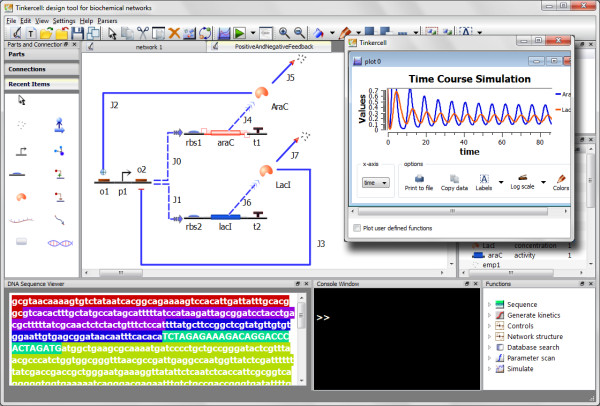
**Synthetic Biology Example**. Shown here is a screenshot of an oscillator constructed using positive and negative feedback. The network design is the same as the one shown in Figure 9, but the difference is in the way the network is constructed. This network is constructed by connecting parts together; the kinetics is based on the properties of the parts, thus loading parts from a database would affect the network dynamics. This particular network has been constructed in *E. coli *and shown to oscillate [9]. Both genes, araC and lacI, are regulated by the same promoter, p1. The promoter is regulated by the proteins, AraC and LacI, thus forming the feedback loop. The dotted connection between the promoter region and the two genes indicates that the promoter p1 is situated upstream of both genes. Therefore, it is implied that there are two copies of the promoter in the physical DNA. The dotted lines from the gene to the proteins represent multiple reactions, which is meant to capture transcription, translation and protein folding stages, all of which contribute to the delay that is required for the oscillation. The bottom left corner shows the sequence of one of the contiguous sequences of DNA, starting from the promoter region and ending with the araC gene. A video demo showing how this network is constructed is available online at  under the "Demos" link.

The second method of modeling gene regulatory networks involves the same equilibrium assumption mentioned above. However, the entire gene is not represented as a single item; it is split into a set of distinct parts. Each part can be moved individually. The rate expression can be described in terms of the promoter strength and RBS strength, thus allowing a user to swap one part with another, e.g. replace a weak RBS with a strong one. The rate expression is defined in terms of the parts that are directly upstream of the coding region, so swapping parts would automatically update the rate expressions. Figure 10 shows the same network as the one in Figure 9 but using distinct parts such as promoters, RBS, protein coding regions, and terminators.

#### Explicitly defining intermediate steps

A more elaborate way of describing gene regulatory networks is to define each reaction in the transcription and translation process, including the movement of RNA polymerase across the gene [47]. Creating such a model would be nearly impossible without some sort of automation, and TinkerCell provides a function for automating this process. While such models can avoid the use of equilibrium assumptions, simulation can be time-consuming due to the fact that each transcription and translation step is comprised of multiple reactions. But this method can often capture differences in behavior due to delays or stochastic fluctuations in the intermediate stages of transcription and translation [47].

### List of functions provided through the C and Python interface

While the visual side of TinkerCell provides the interface for constructing a model, the analyses are carried out by C and Python functions. All of the C programs use the TC_api.h header file in order to interact with TinkerCell's visual interface. New C programs can be added to TinkerCell by building them as dynamic libraries. Python scripts can be added to TinkerCell by listing the script name and description in the "pythonscripts.txt" file. All C and Python functions automatically run on separate threads, allowing users to continue working as the program is running and utilizing the multi-core architecture of most modern computers.

#### Fast simulators

The default simulators use existing C libraries that perform deterministic and stochastic simulation. For example, the stochastic simulator uses a custom Gillespie [35] C library available from evolvenetworks.sourceforge.net. The C library accepts a user-defined stoichiometry matrix and propensity function. The Gillespie simulator in TinkerCell takes the stoichiometry matrix and reaction rates from the TinkerCell model and generates the C code containing the stoichiometry matrix and propensity function. This C code is compiled and linked against the existing Gillespie algorithm library. The compiled C program performs the simulation and outputs the result to TinkerCell's plot window. Therefore, the simulation itself is performed by a compiled C program, which means that the simulation will be fast. Many conventional simulators interpret the rate equation string and construct an internal representation of the equation, which is used to compute the rate value during every iteration; this can be an order of magnitude slower than the compiled program. The same idea is used by the deterministic simulation, which uses the Sundials CVODE numerical integrator [34] to perform the simulation. One drawback is that there is a short time delay when compiling the code, which may be more than the time to simulate simple networks. Therefore, the speed gain due to this strategy is only visible for larger networks and time consuming stochastic stimulations. However, this demonstrates how existing C libraries can be used without modification to create new simulators for TinkerCell. The Tiny C Compiler [48] and GNU C Compiler [49] are used to perform the compilation in MS Windows and Unix based systems (including Mac), respectively.

Further, the simulation programs use a function called "createInputWindow" that is provided by the API. This function allows C and Python programs to create simple dialogs where the user can input parameters such as the time for simulation or the type of simulation. There are several such functions that allow C and Python programs to interact with the user through visual interfaces.

#### Two-parameter steady state analysis

Among the functions included with TinkerCell are one-parameter and two-parameter steady state analyses. Both functions are loaded as C plug-ins, and they use the Sundials CVODE integrator [34]. The two-parameter steady state analysis plots a 3D surface plot, showing the value of a target variable as a function of two parameters in the system.

#### Visual inputs and outputs for C and Python programs

While C and Python programs are generally expected to take command-line inputs and produce command-line outputs, TinkerCell provides functions that allow C and Python functions to interact with the user directly. TinkerCell's C and Python API contain functions for displaying dialogs with input tables. The outputs from these dialogs are returned to the calling C or Python program. The C or Python program can then use the information from the model and the inputs from the user to perform some analyses. As an output, the C and Python programs can change the size or color of items in the model, circle items in the model, or display numbers or strings next to items in the model. At the same time, the programs may also print to the TinkerCell command-line window.

##### Flux balance analysis

The flux balance analysis is one example where a C program produces visual outputs. This particular program uses a C++ plug-in to create a custom input window. The C++ plug-in sets up the windows, tables, and buttons specifically for flux balance analysis. This window simply serves as a user interface to a C program. It receives input from the C++ plug-in and uses the LPSolve C library [36] to do the optimization. The C program then changes the width of the reaction arcs according to the result from LPSolve. The optimal fluxes are also displayed next to the reactions arcs (see Figure 3).

##### Sensitivity analysis

The sensitivity analysis is yet another example of visual output (see Figure 3). The senstivity converts the TinkerCell model into a PySCeS [25] model and uses PySCeS to perform the analysis. The script then takes the PySCeS output and colors the reactions in the network according to their control coefficients.

##### Accessing *E. coli *genetic parts through RegulonDB

RegulonDB [44] houses a large set of promoters, transcription factor binding sites, and RBS for the *E. coli *K12 strain. Through a Python script, a user can load sequences and other information such as promoter type into TinkerCell models. The script provides the user with a visual interface for selecting the parts. The script also limits the search by using known information. For example, if the transcription factor LacI is repressing a promoter, then the script will show only those binding sites that are targeted by LacI (see Figure 4), allowing a user to automatically generate the correct network using real parts. The Python script is able to use the family information of objects in the model to identify whether it is a promoter, RBS, coding region, or transcription factor. It would be impossible to search for an appropriate part with this information in the model, which demonstrates how the meta-data is useful for database search.

#### Automated kinetics

One of TinkerCell's objectives is to combine the appeal of visual network design with the flexibility of programming. Automatic generation of kinetics is a good example where this objective is met. There are three functions currently included with Tinker-Cell that fall in this category. First, the "Hill equation" Python function automatically generates the fractional saturation model for transcription regulation based on the activators and repressors of a promoter. Second, the "binding reactions" C program automatically generates all the intermediate stages of a protein that form multiple complexes. Third, the "multiple step process" function can automatically insert intermediate steps into any given reaction. For example, if the conversion of one molecule to another requires several intermediate stages, this function can be used to automatically generate those intermediates reactions. Gene regulatory networks are one of the areas where this function can have additional relevance.

##### Automatically generate multiple conformations of a protein

Consider a simple situation of two small proteins binding to a larger protein. In addition to the three proteins, there are three other complexes. However, the number of possible complexes grows exponentially with the number of binding proteins, thus modeling every complex explicitly becomes impractical [50]. Generally, a modeler may choose to ignore such details due to the difficulty in modeling. TinkerCell offers a feature for automatically generating all necessary combinations. This feature is provided by an existing C function that recursively generates all the intermediate states and reactions. Integrating the existing function into TinkerCell required three steps: obtaining "Binding reactions" in the set of items selected by the user, getting the stoichiometry matrix for those reactions, and passing the stoichiometry matrix to the existing C function. The output from the C function is then used to modify the TinkerCell model.

##### Automatically generate intermediate stages of a multi-step process

Another automated feature is converting a single reaction into a multi-step reaction. This feature was inspired by the fact that transcription can be understood as several individual reactions, where each reaction is catalyzed by RNA polymerase. Modeling genetic networks in this manner can have significantly different outcomes in some situations [47]. It would be impossible for a user to draw hundred of reactions for each transcriptional process in a model. TinkerCell offers an automated function for converting a single process into a multistep process. The visual model remains unchanged, but the selected reactions will represent multiple reactions. While an alternative approach might be delayed differential equations, this approach allows other details such as leaks in transcription and different delays based of the type of process and stochastic simulations.

### TinkerCell file format

TinkerCell stores the entire model as a single XML file, which contains both the model information as well as the graphical information. All the graphical objects in TinkerCell are generated from XML files, and therefore they can be saved as XML files. The model itself is stored as a set of components and information associated with those components. The information associated with each component is a set of tables containing the parameters, reaction rates, function definitions, events, DNA sequence, annotation, and any other information pertaining to that component in the model. The file format is therefore just a list of objects, their family information, and data tables associated with the objects. The datatables are labeled as "parameters", "reaction rates", "text attributes", and so on. The format is generic, which reflects TinkerCell's underlying structure.

### Example: dual feedback synthetic oscillator

Genetic circuits can be built in TinkerCell by snapping genetic parts together. The regulatory rates will be automatically assigned when promoters and RBS are connected upstream of coding segments. Delays in transcription and translation can be explicitly modeled by introducing intermediate steps automatically. These features are used effectively in the synthetic genetic circuit shown in Figure 10, which is a dual feedback oscillator that has been constructed in *E. coli *[9]. Video demonstrations for the construction of this synthetic circuit and two other circuits are also provided under the "Demos" link at . The videos are also available through YouTube using "TinkerCell" as the search keyword. The demonstration also shows how to load sequences from RegulonDB.

## Conclusion

We have described an application called TinkerCell that combines the flexibility of programming with a visual interface. In addition, TinkerCell provides the structure for supporting standards and exchange of information. TinkerCell is not intended to be yet another modeling application. TinkerCell is an application for bringing together models, information, and algorithms. Therefore, it serves a host to C and Python programs that provide functions useful for biological engineering. The API has been demonstrated to be very versatile because numerous C and Python programs and packages have been readily integrated into TinkerCell.

The modular modeling framework of TinkerCell can serve two purposes. The first is to aid in the engineering process by allowing one modeler to use existing modules to design new networks. The second purpose is that modeling with modules can be used to explore the possible implication of functional modules in biology. It is an open question whether a module's functionality is retained when it is placed in different situations [39]. Answers to such questions will be highly relevant to synthetic biology, since the ability to construct one system using another is a central theme in engineering. TinkerCell can serve as a platform for testing how different modules behave and what types of modules are able to retain their functional identity. The flexible modeling framework provides such experiments to be carried out at different levels of detail. For example, some issues regarding modularity may not be visible when using fractional saturation models of gene regulation, but they may be evident when the reactions are modeled explicitly. Since TinkerCell provides different ways of modeling, it is suitable for studying such questions.

### Work in progress

TinkerCell is continually being updated. In particular, we are currently working in four areas, which include:

• better use of community standards and tools such as the Systems Biology Workbench [19,22,51] and the Synthetic Biology Open Language standards [18]

• addition of more analysis functions from PySCeS [25] such as bifurcation analysis and parameter scans

• better integration between the Antimony scripting language [32] and the visual representation to allow text and graphical views to be easily interchanged

• the ability to build models with poorly defined parts.

The last feature needs more explanation. Parameters describing the complete dynamics of a given biological part are rarely known. To address this reality, TinkerCell would be able to define parameters with an upper and lower limit or a standard deviation value, indicating the uncertainty of the parameter value. The ability to include uncertainly measures on parameters will allow us to develop algorithms to determine the robustness of a variety of designs in the face of parameter variation. The most robust designs could then be selected for construction in vivo.

Integrating future synthetic biology standards [18] would lead to better database integration, which would allow TinkerCell to provide some useful features for experimentalists. These include:

• ability to list parts that are compatible with each other, where compatibility includes physical assembly as well as known interferences or cross-talk between the parts

• conveniently providing references and user ratings when they are available for parts

• estimating standard measurements [16] on parts from experimental data and submitting the information back to a database

The above features are being developed along with database efforts.

### Platform availability

The TinkerCell application can be successfully compiled and run on most major platforms (Windows, Mac, Linux). Installers are currently available for Windows systems. Installers for Mac will be available in the near future.

## Competing interests

The authors declare that they have no competing interests.

## Authors' contributions

DC was the primary designer and developer for the TinkerCell application. FTB and DC worked on a similar project, Athena [21], which served as a starting point for TinkerCell. HMS provided critical feedback and suggestions during the development of TinkerCell.

## Supplementary Material

Additional file 1 **Plug-in descriptions and sample Python scripts**. A short description of some of the main plug-ins that are available with TinkerCell and a few short scripts showing how the Python language can be used to interact with TinkerCell's visual interface. Click here for file
