# Supplementary material for: TinkerCell: modular CAD tool for synthetic biology
Source: J Biol Eng. 2009 Oct 29;3:19. doi: 10.1186/1754-1611-3-19 (PMC2776589; doi:10.1186/1754-1611-3-19)
Supplement: Additional file 1 — Plug-in descriptions and sample Python scripts. A short description of some of the main plug-ins that are available with TinkerCell and a few short scripts showing how the Python language can be used to interact with TinkerCell's visual interface. [file 1754-1611-3-19-S1.pdf]

Division of labor between the Core library and the plug-ins

The Core library

- Drawing of parts that are defined in a XML format
- Drawing of connections and saving connections to an XML file
- Selecting, inserting, copying, pasting, and moving items on the canvas
- Undo and redo capabilities along with a history window
- Memory management for parts and connections
- Loading C libraries as separate threads (i.e. parallel processing)
- Indexing variables in a model, allowing easy look-up
- Parsing math formulas using muparser library (muparser.sourceforge.net)
- Console window that plug-ins can use for console-based interaction

The plug-ins provide the following features:

Parts Tree Provides a window displaying all the parts listed in an XML file.

Connections Tree Provides a window displaying all the connections listed in an XML file. Each connection is restricted to certain types of parts. For example, a "transcription regulation" can only be made between a "transcription factor" and a "regulator" part.

Part Insertion Tool Monitors the Parts Tree and allows user to insert parts from the tree.

Connection Insertion Tool Monitors the Connections Tree and allows user to make permitted connections.

Selection Tool Highlights selected items and handles a few other user interface details.

Name and Family Tool Displays the family information of items and allows users to change the family of items.

Collision Detector Detects collision events and reports to other plug-ins. This information is used by several other plug-ins to respond when user places one item adjacent to another.

Container Tool Allows user to drag items into a container or module. Also displays the parameter window.

Plot Tool Provides a graphing window that can handle multiple graphs. C and Python programs can output to the graph window directly.

3D Plots Provides function for plotting 3D surface plots inside the Plot Tool's graph window.

Module Tool Allows construction of modules with interfaces that can be connected.

Gene Regulatory Network Tool Provides various automated features that allows easier construction of genetic networks. For example, when a user places a promoter upstream of a gene, the gene's transcription rates are automatically updated. Similarly, when a user connects a transcription factor to a regulator, the kinetics are automatically generated.

Antimony Tool Uses libAntimony (antimony.sourceforge.net)

Sample Python scripts

Linear algebra operations on the Stoichiometry matrix

The following script gets the list of reactions that are selected by the user and generates a stoichiometry matrix for just the selected reactions. It then uses SciPy to perform QR and LU factorization on the stoichiometry matrix. Most of the output is not shown for reading clarity.

```
>>A = selectedItems()
>>N = stoichiometry(A)
>>print N[0]

('s1', 's2', 's3', 's4', 's5')

>>print N[1]

('J0', 'J1', 'J2', 'J3', 'J4', 'J5')

>>M = matrix(N[2])
>>print M

[[-1.  0.  0.  0.  1.  0.]
 [ 1. -1.  0.  0.  0.  0.]
 [ 0.  1. -1.  0.  0.  1.]
 [ 0.  0.  1. -1.  0.  0.]
 [ 0.  0.  0.  1. -1. -1.]]

>>from scipy import linalg
>>print linalg.qr(M)
>>print linalg.lu(M)
```

Get all the part sequences in the model as a multi-FASTA file

The following script will find all the components in the model that belong to the family called “DNA”, which is the root family for promoters, RBS, genes, and other parts that belong on the DNA. Then the script will get the sequence for each part and print the sequence to the screen in FASTA format.

```
import pytc
A = pytc.itemsOfFamily('DNA');

if (len(A) > 0):
  names = pytc.getNames(A);
  attribs = ('sequence',);
  seqs = pytc.getAllTextNamed(A,attribs);

  n = len(seqs);
  s = '';
  for i in range(0,n):
    s += '>' + names[i] + '\n' + seqs[i]
       + '\n';

  print s;
```

Getting annotation and sequence information about a module and its sub-components

The following script will find a module with a particular name and obtain all of its sub-parts. Then it will loop through the sub-part and print annotation information and sequence for each sub-part. Names of people in the output have been replaced with “no name”, and some of the longer sequences have been truncated.

```
>>part = find("BBa_I0462")
>>print annotation(part)
('no name', '12/04/2003',
'luxR Protein Generator', 'uri', 'reference')

>>print getTextAttribute(part,"format")
BBa

>>print getTextAttribute(part,"type")
composite

>>subparts = getChildren(part)
>>for i in subparts:
       print getName(i) + " is a " + getFamily(i);
BBa_I0462_B0012 is a Terminator
```

```
BBa_I0462_B0010 is a Terminator
BBa_I0462_C0062 is a Coding
BBa_I0462_B0034 is a RBS

>>for i in subparts:
       print getName(i) + " : ";
       print annotation(i);
BBa_I0462_B0012 :
('no name', 'NA',
'Transcription terminator for the E.coli RNA
polymerase.', 'uri', 'reference')

BBa_I0462_B0010 :
('no name', '11/19/2003',
'Transcriptional terminator consisting of
a 64 bp stem-loop', 'uri', 'reference')

BBa_I0462_C0062 :
('no name', '2003', 'luxR repressor/activator',
'uri', 'reference')

BBa_I0462_B0034 :
('no name', '2003', 'RBS based on
Elowitz repressilator', 'uri', 'reference')

>>for i in subparts: print getName(i) + " : ";
          print getTextAttribute(i,"sequence");
BBa_I0462_B0012 :
tcacactggctcaccttcgggtgggcctttctgcgtttata

BBa_I0462_B0010 :
ccaggcatcaaataaaacgaaaggctcagtcgaaagact
gggcctttcgttttatctgttgtttgtcggtgaacgctctc

BBa_I0462_C0062 :
aaagaggag ... ttctgcgtttata

BBa_I0462_B0034 :
aaagaggagaaa

>>print
    getParameter(subparts[3],"strength")
5.0
```
